# Optimal and safe standard doses of midazolam and propofol to achieve patient and doctor satisfaction with dental treatment: A prospective cohort study

**DOI:** 10.1371/journal.pone.0171627

**Published:** 2017-02-09

**Authors:** Rikuo Masuda, Mutsumi Nonaka, Akiko Nishimura, Kinuko Gotoh, Shuichirou Oka, Takehiko Iijima

**Affiliations:** Department of Perioperative Medicine, Division of Anesthesiology, Showa University School of Dentistry, Tokyo, Japan; UNITED KINGDOM

## Abstract

**Background:**

The incidences of morbidity and mortality caused by pharmacosedation for dental treatment have not yet reached zero. Adverse events are related to inappropriate respiratory management, mostly originating from an overdose of sedatives. Since sedation is utilized for the satisfaction of both the dentist and the patient, the optimal dose should be minimized to prevent adverse events. We attempted to define the optimal doses of midazolam and propofol required to achieve high levels of patient and dentist satisfaction.

**Methods:**

One thousand dental patients, including those undergoing third molar extractions, were enrolled in this study. A dose of 1 mg of midazolam was administered at 1-minute intervals until adequate sedation was achieved. Propofol was then infused continuously to maintain the sedation level. Both the patients and the dentists were subsequently interviewed and asked to complete a questionnaire. A multivariate logistic regression analysis was used to examine the factors that contributed to patient and dentist satisfaction.

**Results:**

The peak midazolam dose resulting in the highest percentage of patient satisfaction was 3 mg. Both a lower dose and a higher dose reduced patient satisfaction. Patient satisfaction increased with an increasing dosage of propofol up until 4 mg/kg/hr, reaching a peak of 78.6%. The peak midazolam dose resulting in the highest percentage of dentist satisfaction (78.8%) was 2 mg. Incremental propofol doses reduced dentist satisfaction, in contrast to their effect on patient satisfaction. The strongest independent predictors of patient satisfaction and dentist satisfaction were no intraoperative memory (OR, 5.073; 95% CI, 3.532–7.287; *P*<0.001) and unintentional movements by the patient (OR, 0.035; 95% CI, 0.012–0.104; *P*<0.001), respectively. No serious adverse events were reported.

**Conclusion:**

We found that 3 mg of midazolam and 3 mg/kg/hr of propofol may be the optimal doses for maximizing both patient and dentist satisfaction. Although this level of sedation is relatively light, memory loss and an absence of unintentional patient movements can be expected without adverse events.

## Introduction

Intravenous sedation has been widely used for dental treatment. Although the incidences of morbidity and mortality are reportedly low [1], undesirable outcomes have been occasionally reported [2]. The intra-oral procedure itself tends to prevent the patient from feeling drowsy; therefore, the anesthesia-provider tends to use sedatives excessively. Overdoses of benzodiazepine occasionally cause agitation or lethal respiratory suppression. The sensitivity to sedatives may also vary between individuals, possibly leading to an overdose or the excessive use of multiple drugs [2].

The aim of this study was to determine whether a universal dose of sedatives for optimal sedation or a dose sufficient to achieve ideal levels of anesthetic parameters (e.g., no intraoperative memory, no patient movement) could be identified.

Our study examined whether an optimal dose could be determined and whether the required dose for individuals may vary using a statistical analysis based on data from a large cohort. Our study cohort ranged from mostly patients receiving conscious sedation to those receiving deep sedation. The applied surgical interventions included third molar extractions, which usually require general anesthesia or deep sedation; therefore, we were able to examine the indications for conscious sedation even for relatively invasive procedures.

## Materials and methods

### Design

**Fig 1:**
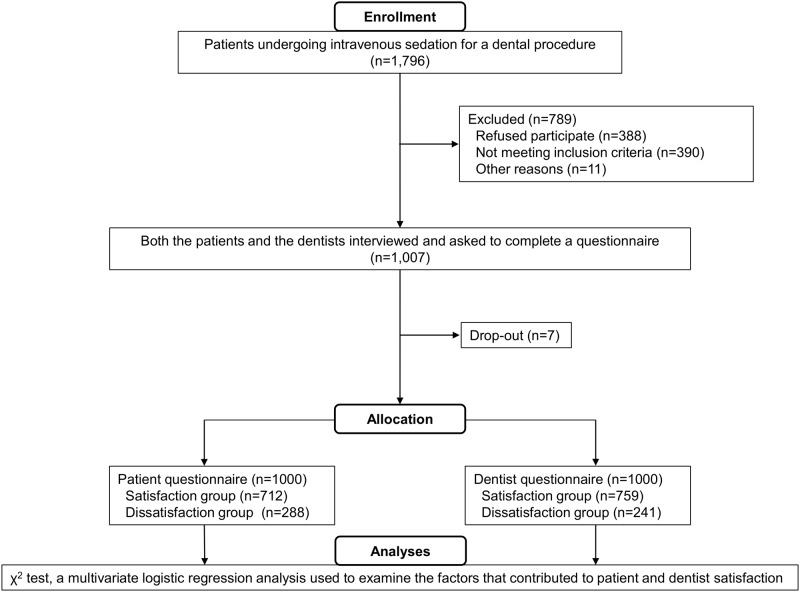
Flow chart showing enrollment, study inclusion, and data analysis.

We designed a prospective cohort study (Fig 1) to define the optimal doses of midazolam and propofol required to achieve high levels of patient and dentist satisfaction. This study was approved by the Clinical Research Ethics Committee of the Showa University School of Dentistry (Approval number; 2011–019) and was registered at UMIN-CTR (UMIN000014228). The ethics committee approved the study on September 20, 2011, and patient recruitment and follow-up were performed from January 27, 2012, to April 25, 2013. We also registered our protocol with the UMIN system (a nationwide registry) in 2014. At our university, registration with UMIN was not a requirement in 2011. Therefore, we did not register with UMIN before the start of the clinical trial. UMIN subsequently approved our trial even though the clinical study had ended. The full trial protocol is available in the Supporting Information section (S1 Protocol).

### General setting

The enrolled patients had an American Society of Anesthesiologists (ASA) class of 1–2 and were 20 years or older; all the patients underwent intravenous sedation for a dental procedure. Most of the patients had a dental phobia or an unintentional gag reflex. Patients with allergies to midazolam or propofol, dementia, intellectual disability, or cerebral palsy were excluded from the study. Written informed consent to participate in a clinical study was obtained from all the patients prior to the start of the procedure.

All the procedures were performed under intravenous sedation in the outpatient department or in an operating room at the Showa University Dental Hospital (Tokyo, Japan). Patients were instructed to refrain from eating food for 6 hours and from drinking clear liquids for 2 hours prior to sedation. Premedication was not performed in any of the cases. The patients’ vital signs (pulse oximetry and non-invasive blood pressure) were monitored. This protocol represents the monitoring standards required for patient safety [3]. The patients’ vital signs were measured every 5 minutes during sedation, treatment, and recovery.

### Intravenous sedation methods

Intravenous sedation was provided by members affiliated with the Division of Anesthesiology, Department of Perioperative Medicine, Showa University, School of Dentistry, who had been trained for anesthesia management, including general anesthesia. The provider was supervised by a“dental anesthesiologist” certified by the Japanese Society of Dental Anesthesiologists in Japan. An intravenous cannula was inserted into a suitable vein, and a continuous infusion of crystalloid (Soldem^®^ 1; Terumo, Tokyo, Japan) was started. First, 1 mg of midazolam was given, and an additional bolus of 1 mg every minute was allowed until adequate sedation was achieved. The sedation was judged as satisfactory once the patient began to exhibit slurred speech and/or ptosis of the eyelids. The midazolam dose required to introduce sedation before the start of propofol administration was defined as the initial dose of midazolam (mg). After induction with midazolam, sedation was maintained using a continuous infusion of propofol throughout the procedure, which has shorter context-sensitive half-life than midazolam. The propofol infusion was started after confirming the optimal level of sedation using midazolam and when the surgical procedure was about to begin. The propofol dose was determined so as to maintain a sedation level at which the patient could be roused by verbal commands or physical stimulation. Ultimately, the dose depended entirely on the discretion of each dental anesthesiologist, after taking the duration of the procedures and the operative stress into consideration. The propofol rate required to maintain sedation at the start of propofol injection was defined as the initial dose of propofol (mg/kg/hr). A change in the propofol dose and the addition of midazolam were not fundamentally allowed. However, supplemental sedation according to the patient’s condition (movement, phonation, etc.) was allowed using a bolus injection (propofol or midazolam) or by increasing the propofol rate, if necessary. The quantity and timing of the bolus injection and the increased rate of propofol were left to the discretion of the anesthesiologist. On the other hand, if the sedation became too deep, the rate of propofol was decreased. The amounts of midazolam and propofol that were used as of the end of the procedure were defined as the total doses of midazolam and propofol, respectively. The dental treatment was started after an assessment of the sedation level using the Observers Assessment of Alertness/Sedation (OAA/S) Scale [4]. Local analgesia (2% lidocaine with 1: 80,000 adrenaline) was administered as necessary. Patients were given oxygen via a nasal cannula at a rate of 2–3 L/min, as needed.

After the procedure, the patients were kept calm until recovery. The patients were required to achieve the following three conditions before they were discharged from hospital: (1) patients had to have a clear sensorium and to be able to respond appropriately to verbal questioning; (2) the patient’s vital signs (blood pressure, pulse rate, and oxygen saturation) had to be almost within the same range as their baseline values; and (3) the patients had to be able to walk without staggering.

### Questionnaire

Patient comfort (rated as good, fair, poor or bad) and intraoperative memory (rated as none, a little, most, or all) were assessed after permission for hospital discharge had been granted. “Patient satisfaction” was defined as a patient comfort rating of “good.” “No intraoperative memory” was defined as the complete absence of any memory of the procedure.

After each procedure, the dentists were asked questions regarding the following parameters: patient’s mouth opening, patient’s response, patient’s compliance (rated as good, fair, poor, or bad), unintentional movements (rated as sizable, frequent, acceptable, or none), and ease of performing the procedure (rated as good, fair, poor, or bad). “Dentist satisfaction” was defined as an ease of performing the procedure rating of “good.” “Unintentional movements” were defined as the presence of a gag reflex, coughing, or excessive movements during sedation. The patient’s mouth opening and the patient’s responses were measured using a 3-point scale (rapid, slow, or poor).

### Sample

The sample size calculation for this study was performed using G*Power V.3.1.9.2. We conducted a pre-survey to determine the sample size. The sample size for the χ^2^ test so as to detect significant differences in the effects on patient satisfaction and dentist satisfaction between each sedative dose level (midazolam, 4 groups; propofol, 3 groups) were calculated. First, 50 participants were recruited to determine each effect size. For patient satisfaction, 128 and 155 patients were required to detect a difference between the midazolam dose level and the propofol dose level, respectively (power of 80% and α set at 0.05). For doctor satisfaction, 3,241 and 295 patients were required to detect a difference between the midazolam dose level and the propofol dose level, respectively (power of 80% and α set at 0.05). After 1,000 participants, we performed a post-hoc power analysis. The observed powers of patient satisfaction were 0.89 and 0.66 for the midazolam dose level and the propofol dose level, respectively (effect size at 0.12 and 0.09, α set at 0.05). The observed powers of dentist satisfaction were 0.97 and 0.98 for the midazolam dose level and the propofol dose level, respectively (effect size at 0.145 and 0.142, α set at 0.05). Although the power for the statistical analysis comparing patient satisfaction and the propofol dose was less than 0.8, a dose level of more than 2 mg/kg/hr of propofol did not appear to have a significant impact on patient satisfaction. Therefore, we believed that 1,000 participants were sufficient and stopped patient recruitment.

### Statistical analysis

Values for continuous variables were expressed as the mean ± standard deviation (SD) and were compared between the satisfied and dissatisfied groups using a two-sample *t*-test. Nominal variables were presented as the percentage (%) and were analyzed using the χ^2^ test. Factors putatively contributing to the patient and doctor satisfaction were selected if they had a *P* value of less than 0.2 when analyzed using simple logistic regression. These factors were then entered into a multivariate logistic regression analysis (forward selection, maximum likelihood ratio method). We analyzed the relationships between patient or doctor satisfaction and the initial dose of midazolam or propofol using the χ^2^ test. The most significant dose was selected as that with an adjusted residual > 1.96. We also analyzed the dependency of the most significant predictor of patient or doctor satisfaction as selected using the χ^2^ test. The relationships between supplemental sedation and a lower propofol dose and patient or dentist satisfaction were analyzed using the χ2 test. A *P* value of less than 0.05 was considered significant. All the statistical analyses were performed using IBM^®^ SPSS^®^ Statistics software (version 20).

## Results

### Patient backgrounds

The final number of participants was 1,000 (outpatients: 966, inpatients: 34), and women accounted for 64.2% (Fig 1, Table 1). A total of 78.8% of the patients were classified as ASA 1, and all the patients tolerated sedation well with no serious adverse effects or complications. The reasons for the sedation were as follows: dental phobia, 51.7%; invasive treatment, 31.7%; unintentional gag reflex, 10.1%; systemic illness, 1.2%; and others, 5.3%. The types of therapy were as follows: surgical treatment including tooth extraction, 58.9%; conservative dental treatment, 24.7%; dental implant placement, 15.8%; and prosthodontics, 0.6%.

**Table 1:** Patient backgrounds.

Variable	Value
n	1,000
Sex, male/female	358/642
Age, years	40 ± 14 (20–85)
Body height, cm	163 ± 8 (142–184)
Body weight, kg	59 ± 12 (33–102)

Values are number of individuals or mean ± standard deviation (min-max).

### Intravenous sedation

The number of patients in each group of initial midazolam dose were as follows: 1 mg, 40 patients; 2 mg, 354 patients; 3 mg, 482 patients; 4 mg, 107 patients; and over 5 mg, 17 patients. The number of patients in each group of initial propofol dose were as follows: 1 mg/kg/hr, 26 patients; 2 mg/kg/hr, 531 patients; 3 mg/kg/hr, 401 patients; and over 4 mg/kg/hr, 42 patients. The mean initial dose of midazolam was 2.7 ± 0.8 mg (range, 1–7 mg). and the mean initial dose of propofol was 2.5 ± 0.6 mg/kg/hr (range, 1–5 mg/kg/hr). The median OAA/S scale before the start of therapy was 4 (lethargic response to name spoken in a normal tone). The mean total dose of midazolam was 2.8 ± 0.8 mg (range, 1–7 mg), and the mean total dose of propofol was 132.5 ± 75.3 mg (range, 15–530 mg). Supplemental sedation was performed for 213 cases. The reasons for the supplemental sedation were as follows: patient's complaint, 67.1%, dentist's complaint, 14.1%, and dental anesthesiologist’s judgment (patient's gag reflex, hypertension, etc.), 18.8%. The median score level for the OAA/S scale during treatment was approximately 4, which was regarded as average. The mean duration of the procedures was 44 ± 27 min (range, 1–205 min), the mean duration of sedation was 61 ± 29 min (range, 10–225 min), and the mean duration until recovery was 15 ± 7 min (range, 3–55 min). No serious adverse events or deaths were recorded during the study.

### Questionnaire

More than 70% of the patients (71.2%) and more than 75% of the dentists (75.9%) selected the “good” response when asked about patient and dentist satisfaction in the questionnaire, respectively. Most patients were able to respond and to open their mouths. Intractable unintentional movements rarely occurred. The dental treatments were smoothly completed in most of the cases (Table 2).

**Table 2:** Questionnaire data.

**Patient questionnaire**	
**Intraoperative memory**	
None	393
A little	455
Most	97
All	55
**Patient comfort**	
Good	712
Fair	225
Poor	54
Bad	9
**Dentist questionnaire**	
**Patient mouth opening**	
Rapid (Mouth gag is unnecessary.)	140
Slow (Sometimes mouth gag is necessary.)	697
None (Mouth gag is necessary.)	163
**Patient response**	
Rapid	280
Slow	688
None	32
**Patient compliance**	
Good	723
Fair	197
Poor	72
Bad	8
**Unintentional movements**	
None	597
Acceptable	331
Frequent	63
Sizable	9
**Ease of procedure**	
Good	759
Fair	209
Poor	27
Bad	5

Values are number of individuals. Unintentional movements: gag reflex, coughing, or excessive movements during sedation.

### Patient satisfaction

**Fig 2:**
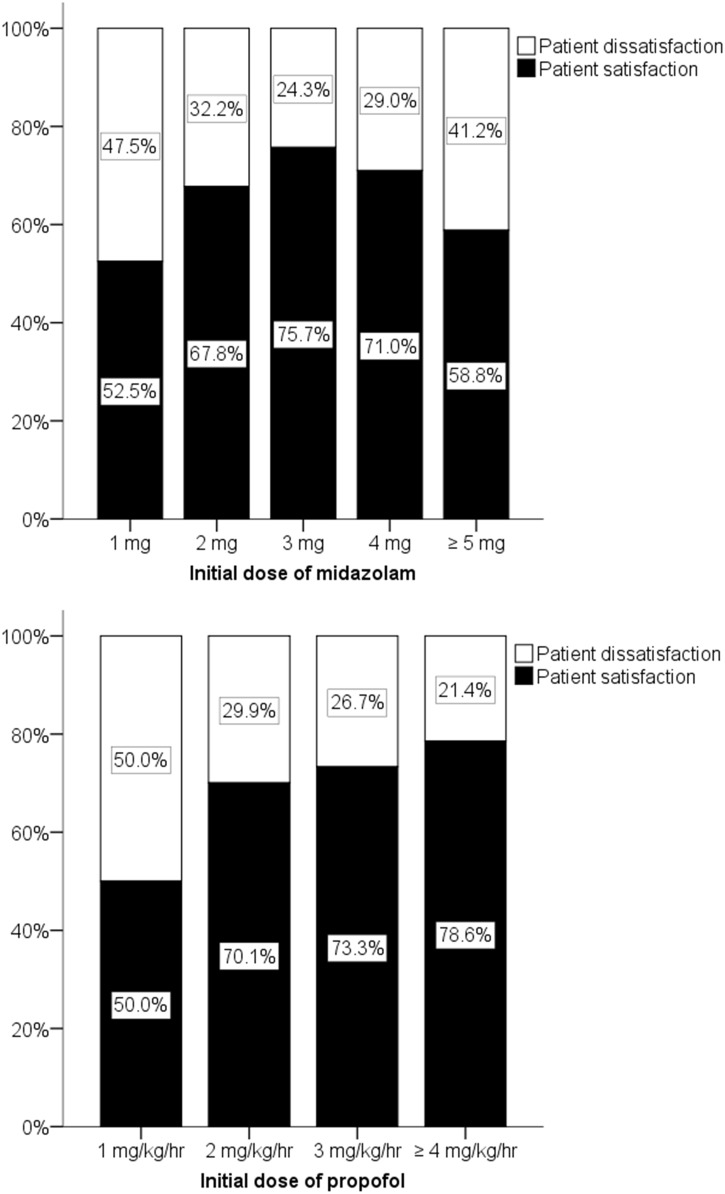
Patient satisfaction according to initial doses of midazolam and propofol. Initial dose of midazolam: midazolam dose required to introduce sedation before the start of propofol administration; initial dose of propofol: propofol rate required to maintain sedation at the start of propofol injection; patient satisfaction: good; patient dissatisfaction: fair, poor, or bad.

**Fig 3:**
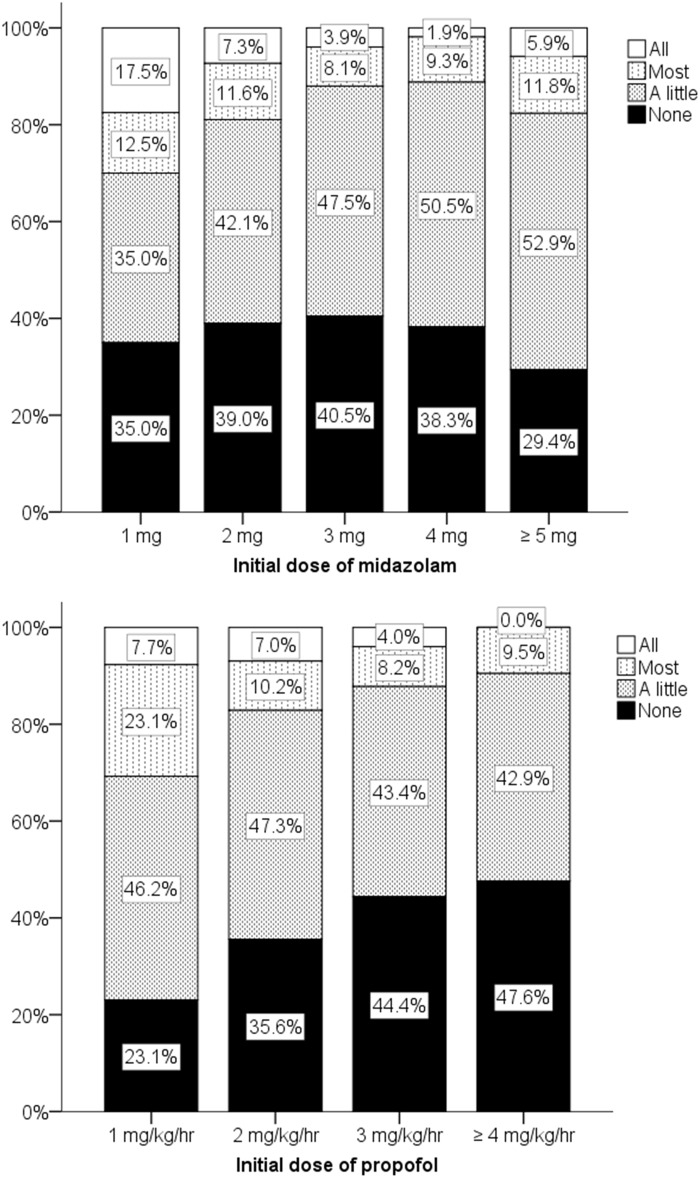
Intraoperative memory according to initial doses of midazolam and propofol. Initial dose of midazolam: midazolam dose required to introduce sedation before the start of propofol administration; initial dose of propofol: propofol rate required to maintain sedation at the start of propofol injection.

No significant differences between patient satisfaction and patient dissatisfaction were seen when analyzed according to sex, age, body weight, initial dose of midazolam, total dose of midazolam, patient mouth opening, patient response, patient compliance, or unintentional movements (Table 3). Significant differences were observed when the patients were analyzed according to the initial dose of propofol, the total dose of propofol, the duration of the procedure, no intraoperative memory, and the reason for the management (Table 3). In a multivariate logistic regression analysis performed after the bivariate analyses, “No intraoperative memory” was the most significant predictor of patient satisfaction (OR, 5.073; 95% CI, 3.532–7.287; *P*<0.001) (Table 4). Both midazolam and propofol were also significant factors, although the odds ratios were relatively low (1.253 and 1.313, respectively), suggesting a dose-dependent comfortable sedative effect (Table 4). The relationship between midazolam and patient satisfaction showed a peak effect depending on the dose (Fig 2). Three milligrams of midazolam produced the highest patient satisfaction (adjusted residual, 3.0; *P* < 0.05, χ^2^ test). Three to four milligrams of midazolam induced the loss of most memory in approximately 90% of the patients (Fig 3), but a significant difference between dose levels was not observed (*P* > 0.05, χ^2^ test). Higher doses of propofol resulted in no intraoperative memory (Fig 3); in particular, the effects of a dose of 3 mg/kg/hr of propofol were significantly different from those of lower doses (adjusted residual, 2.7; *P* < 0.05, χ^2^ test). Supplemental sedation significantly decreased patient satisfaction (adjusted residual, -3.6; *P* < 0.05, χ2 test). A lower propofol dose also significantly decreased patient satisfaction (adjusted residual, -5.5; *P* < 0.05, χ2 test).

**Table 3:** Bivariate analysis of factors associated with patient satisfaction.

Factors		Patient satisfaction (n = 712)	Patient dissatisfaction (n = 288)	*P* value
**Background factors**				
**Sex**	**Male**	**259 (36.4%)**	**99 (34.4%)**	*0*.55*
	**Female**	**453 (63.6%)**	**189 (65.6%)**	
**Age, years**		**40 ± 14**	**41 ± 15**	***0*.*328***†
**Body weight, kg**		**58 ± 12**	**59 ± 12**	***0*.*389***†
**Perioperative factors**				
**Initial dose of midazolam, mg**		**2.7 ± 0.8**	**2.6 ± 0.9**	***0*.*055***†
**Initial dose of propofol, mg/kg/hr**		**2.5 ± 0.6**	**2.4 ± 0.6**	***0*.*021***†
**Total dose of midazolam, mg**		**2.8 ± 0.8**	**2.8 ± 0.9**	***0*.*524***†
**Total dose of propofol, mg**		**126.4 ± 70.9**	**147.3 ± 83.5**	***<0*.*001***†
**Duration of procedure, min**		**42 ± 25**	**50 ± 30**	***<0*.*001***†
**No intraoperative memory**	**No (rated as none)**	**349 (49.0%)**	**44 (15.3%)**	*<0*.001**
	**Yes (rated as a little, most, or all)**	**363 (51.0%)**	**244 (84.7%)**	
**Patient mouth opening**	**Yes (rated as rapid or slow)**	**591 (83.0%)**	**246 (85.4%)**	*0*.35*
	**No (rated as none)**	**121 (17.0%)**	**42 (14.6%)**	
**Patient response**	**Yes (rated as rapid or slow)**	**686 (96.3%)**	**282 (97.9%)**	*0*.202*
	**No (rated as none)**	**26 (3.7%)**	**6 (2.1%)**	
**Patient compliance**	**Yes (rated as good or fair)**	**662 (93.0%)**	**258 (89.6%)**	*0*.073*
	**No (rated as poor or bad)**	**50 (7.0%)**	**30 (10.4%)**	
**Unintentional movements**	**Yes (rated as sizable or frequent)**	**48 (6.7%)**	**24 (8.3%)**	*0*.378*
	**No (rated as acceptable or none)**	**664 (93.3%)**	**264 (91.7%)**	
**Reason for sedation**	**dental phobia**	**377 (52.9%)**	**140 (48.6%)**	*<0*.05***
	**invasive surgery**	**208 (29.2%)**	**109 (37.8%)**	
	**gagging reflex**	**82 (11.5%)**	**19 (6.6%)**	
	**systemic disease**	**7 (1.0%)**	**5 (1.7%)**	
	**others**	**38 (5.3%)**	**15 (5.2%)**	
**Type of surgery**	**surgical procedure**	**420 (59.0%)**	**169 (58.7%)**	*0*.741***
	**implant placement**	**108 (15.2%)**	**50 (17.4%)**	
	**cavity treatment**	**179 (25.1%)**	**68 (23.6%)**	
	**impression taking**	**5 (0.7%)**	**1 (0.3%)**	

Values are number (%) of individuals or mean ± standard deviation.

*Values for "male" or "yes" were analyzed using the χ2 test.

**Values for "no" were analyzed using the χ2 test.

***Values for each of the items were analyzed using the χ2 test.

^†^Values for continuous variables were analyzed using the two sample *t*-test.

Initial dose of midazolam: midazolam dose required to introduce sedation before the start of propofol administration; initial dose of propofol: propofol rate required to maintain sedation at the start of propofol injection; no intraoperative memory: completely no memory of the procedure; unintentional movements: gag reflex, coughing, or excessive movements during sedation.

**Table 4:** Multivariate logistic regression analysis of predictors associated with patient satisfaction.

Predictors	OR (95% CI)	*P* value
No intraoperative memory (no vs. yes)	5.073 (3.532–7.287)	<0.001
Patient compliance (yes vs. no)	1.970 (1.151–3.372)	0.013
Initial dose of propofol (per increasing value)	1.313 (1.017–1.694)	0.036
Initial dose of midazolam (per increasing value)	1.253 (1.037–1.516)	0.02
Total dose of propofol (per increasing value)	0.997 (0.995–0.999)	0.002

Abbreviations: OR: odds ratio, CI: confidence interval. No intraoperative memory: completely no memory of the procedure; initial dose of propofol: propofol rate required to maintain sedation at the start of propofol injection; initial dose of midazolam: midazolam dose required to introduce sedation before the start of propofol administration.

### Dentist satisfaction

**Fig 4:**
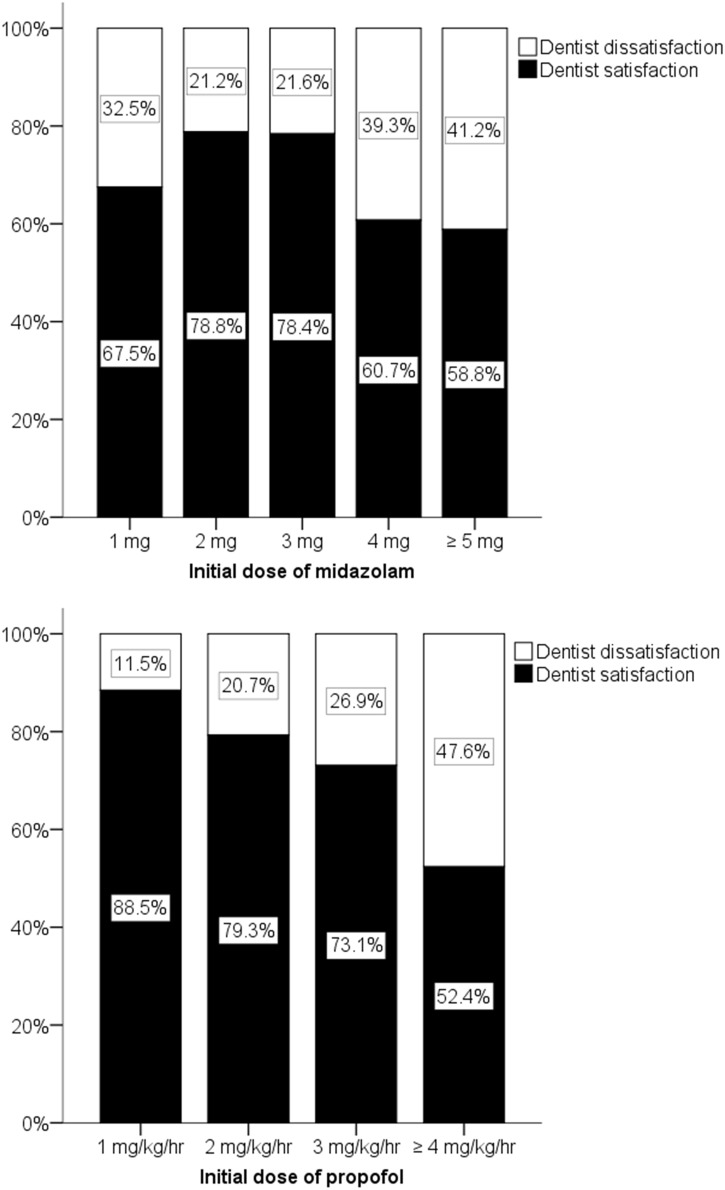
Dentist satisfaction according to initial doses of midazolam and propofol. Initial dose of midazolam: midazolam dose required to introduce sedation before the start of propofol administration; initial dose of propofol: propofol rate required to maintain sedation at the start of propofol injection; dentist satisfaction: good; dentist dissatisfaction: fair, poor, or bad.

**Fig 5:**
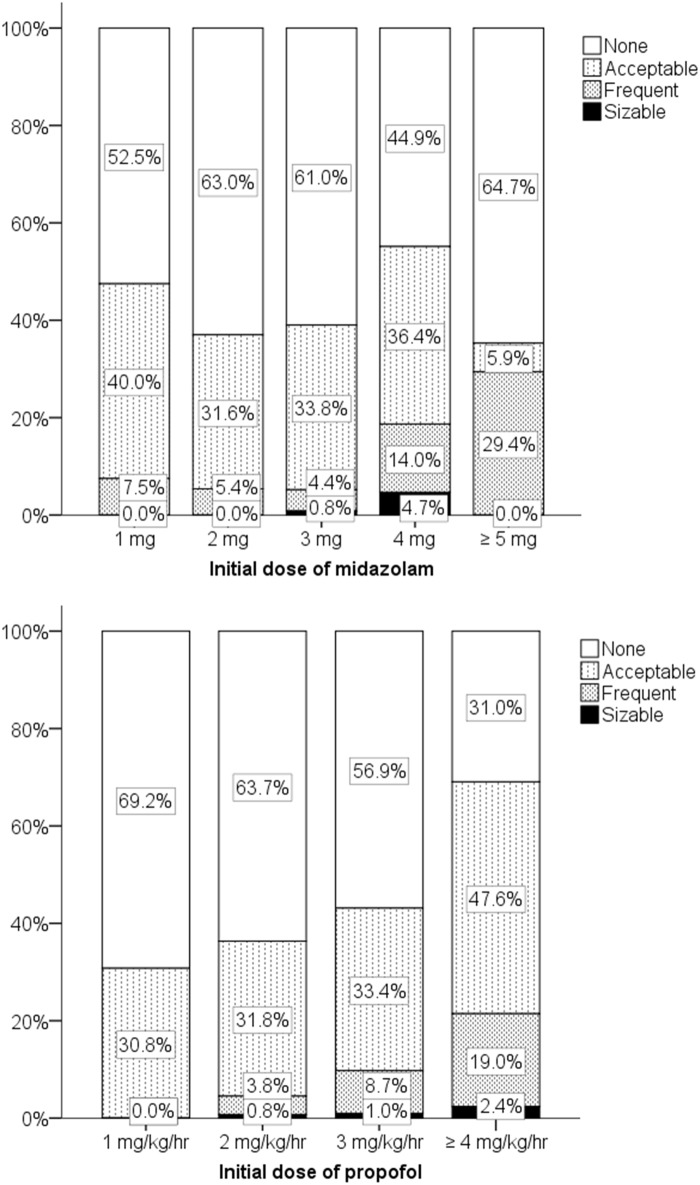
Unintentional movements according to initial doses of midazolam and propofol. Paradoxical reactions: patients' gag reflex, coughing, or excessive movements during sedation; initial dose of midazolam: midazolam dose required to introduce sedation before the start of propofol administration; initial dose of propofol: propofol rate required to maintain sedation at the start of propofol injection.

No significant differences between dentist satisfaction and dentist dissatisfaction were seen when analyzed according to patient age and patient response. Significant differences were observed when analyzed according to patient sex, body weight, initial dose of midazolam or propofol, total dose of midazolam or propofol, duration of procedure, no intraoperative memory, patient mouth opening, patient compliance, and unintentional movements (Table 5). In a multivariate logistic regression analysis performed after bivariate analyses, “Unintentional movements” were the most significant predictor of dentist satisfaction (OR, 0.035; 95% CI, 0.012–0.104; *P*<0.001) (Table 6). The relationship between midazolam and dentist satisfaction showed a peak effect depending on the dose (Fig 4). A midazolam dose of 2–3 mg resulted in dentist satisfaction for more than 78% of the dentists (Fig 4). The number of sizable or frequent unintentional movements increased with initial midazolam doses of over 4 mg, and 3 mg of midazolam produced the lowest frequency of unintentional movements (adjusted residual, 2.4; *P* < 0.05, χ^2^ test). Higher doses of propofol increased the frequency of unintentional movements (Fig 5); in particular, the effects of propofol doses of over 4 mg/kg/hr were significantly different from those of lower doses (adjusted residual, 3.6; *P* < 0.05, χ^2^ test). Propofol doses of over 4 mg/kg/hr were associated with the lowest dentist satisfaction scores (adjusted residual, -3.6; *P* < 0.05, χ^2^ test). Supplemental sedation and a lower propofol dose significantly decreased dentist satisfaction (adjusted residual, -5.5; *P* < 0.05, χ2 test).

**Table 5:** Bivariate analysis of factors associated with dentist satisfaction.

Factors		Dentist satisfaction (n = 759)	Dentist dissatisfaction (n = 241)	*P* value
**Background factors**				
**Sex**	**Male**	**231 (30.4%)**	**127 (52.7%)**	*<0*.001*
	**Female**	**528 (69.6%)**	**114 (47.3%)**	
**Age, years**		**41 ± 15**	**39 ± 14**	***0*.*085***†
**Body weight, kg**		**58 ± 12**	**61 ± 12**	***0*.*001***†
**Perioperative factors**				
**Initial dose of midazolam, mg**		**2.7 ± 0.8**	**2.8 ± 0.9**	***0*.*018***†
**Initial dose of propofol, mg/kg/hr**		**2.4 ± 0.6**	**2.6 ± 0.7**	***<0*.*001***†
**Total dose of midazolam, mg**		**2.8 ± 0.8**	**3.0 ± 1.0**	***<0*.*001***†
**Total dose of propofol, mg**		**122.4 ± 66.8**	**164.2 ± 90.2**	***<0*.*001***†
**Duration of procedure, min**		**42 ± 25**	**51 ± 31**	***<0*.*001***†
**No intraoperative memory**	**No (rated as none)**	**282 (37.2%)**	**111 (46.1%)**	*0*.014**
	**Yes (rated as a little, most, or all)**	**477 (62.8%)**	**130 (53.9%)**	
**Patient mouth opening**	**Yes (rated as rapid or slow)**	**671 (88.4%)**	**166 (68.9%)**	*<0*.001*
	**No (rated as none)**	**88 (11.6%)**	**75 (31.1%)**	
**Patient response**	**Yes (rated as rapid or slow)**	**733 (96.6%)**	**235 (97.5%)**	*0*.472*
	**No (rated as none)**	**26 (3.4%)**	**6 (2.5%)**	
**Patient compliance**	**Yes (rated as good or fair)**	**750 (98.8%)**	**170 (70.5%)**	*<0*.001*
	**No (rated as poor or bad)**	**9 (1.2%)**	**71 (29.5%)**	
**Unintentional movements**	**Yes (rated as sizable or frequent)**	**4 (0.5%)**	**68 (28.2%)**	*<0*.001*
	**No (rated as acceptable or none)**	**755 (99.5%)**	**173 (71.8%)**	
**Reason for sedation**	**dental phobia**	**409 (53.9%)**	**108 (44.8%)**	*<0*.001***
	**invasive surgery**	**240 (31.6%)**	**77 (32.0%)**	
	**gagging reflex**	**61 (8.0%)**	**40 (16.6%)**	
	**systemic disease**	**10 (1.3%)**	**2 (0.8%)**	
	**others**	**39 (5.1%)**	**14 (5.8%)**	
**Type of surgery**	**surgical procedure**	**445 (58.6%)**	**144 (59.8%)**	*0*.942***
	**implant placement**	**119 (15.7%)**	**39 (16.2%)**	
	**cavity treatment**	**190 (25.0%)**	**57 (23.7%)**	
	**impression taking**	**5 (0.7%)**	**1 (0.4%)**	

Values are number (%) of individuals or mean ± standard deviation.

*Values for "male" or "yes" were analyzed using the χ2 test.

**Values for "no" were analyzed using the χ2 test.

***Values for each of the items were analyzed using the χ2 test.

^†^Values for continuous variables were analyzed using the two sample *t*-test.

Initial dose of midazolam: midazolam dose required to introduce sedation before the start of propofol administration; initial dose of propofol: propofol rate required to maintain sedation at the start of propofol injection; no intraoperative memory: completely no memory of the procedure; unintentional movements: gag reflex, coughing, or excessive movements during sedation.

**Table 6:** Multivariate logistic regression analysis of predictors associated with dentist satisfaction.

Predictors	OR (95% CI)	*P* value
Unintentional movements (yes vs. no)	0.035 (0.012–0.104)	<0.001
Patient compliance (yes vs. no)	10.747 (4.851–23.812)	<0.001
Patient mouth opening (yes vs. no)	2.184 (1.400–3.406)	0.001
Sex (male vs. female)	0.628 (0.437–0.903)	0.012
No intraoperative memory (no vs. yes)	0.645 (0.448–0.928)	0.018
Total dose of propofol (per increasing value)	0.994 (0.992–0.997)	<0.001

Abbreviations: OR: odds ratio, CI: confidence interval. Unintentional movements: gag reflex, coughing, or excessive movements during sedation; no intraoperative memory: completely no memory of the procedure.

## Discussion

The sedation level was well maintained within the range of “conscious sedation” in most cases, since patient compliance was well maintained in 93.7% of the patients (including responses of “good” or “fair”), even though some cases of deep sedation were included. Moderate sedation was required in some of the difficult cases. Therefore, this cohort may be a good population for exploring the etiology of unwanted events during conscious and deep sedation. This cohort was also one of the largest ever examined for this type of research. The results of the factorial analysis can thus be considered comprehensive and valid.

Although the most common sedation level was conscious sedation, most of the patients were well satisfied and were discharged from hospital after a period of approximately 15 min without any complications. Deep sedation may not be needed even for invasive procedures, such as third molar extractions. Notably, we had no obvious adverse events in this relatively large cohort. Since adverse events reportedly occurred in 8 out of 1,167 cases [2] and 52 out of 3,320 cases provided by operating surgeons [5], the safety of our practice was most likely due to the low doses of sedatives.

Patients under conscious sedation may recall intraoperative memories if the sedation is too light or they may move unintentionally if the sedation is too deep. These two opposing requirements must be kept in mind when titrating the sedation level. To ensure a loss of intraoperative memory while simultaneously suppressing unintentional movements, the optimal doses of anesthetics should be explored on an individual basis. However, we found that an optimal dose could be fixed even after taking individual variations into consideration. Our study demonstrated that most patients (more than 80%) lost most memory of intraoperative events when they were given even 2 mg of midazolam. A midazolam dose of 2–3 mg may be sufficient to induce the loss of unwanted memories (Fig 3). No intraoperative memory had the strongest impact on patient satisfaction (OR, 5.073; 95% CI, 3.532–7.287; *P*<0.001). A dose of 3 mg of midazolam resulted in a loss of most memory in 88% of the patients. Therefore, we can conclude that 3 mg of midazolam was sufficient to induce a loss of memory in most patients. Unintentional movements appeared to increase if the midazolam dose exceeded 4 mg, and this may explain why a midazolam dose of more than 4 mg reduced not only patient satisfaction, but also dentist satisfaction. Patients may feel poorly if they lose the ability to control themselves, similar to the experience of delirium. Therefore, the ceiling effect of midazolam on patient satisfaction suggests an optimal dose of 2–3 mg. It has been reported that paradoxical reactions induced by intravenous midazolam were often seen if the dose exceeded 4 mg [6, 7]. In a study by Taylor et al. [8], a midazolam dose of 2 mg administered intravenously increased sedation, amnesia, and anxiolytic when administered prior to a propofol infusion for outpatient procedures performed under local anesthesia. Furthermore, even this low dose of midazolam significantly decreased the recollection of uncomfortable intraoperative events without causing adverse intraoperative or postoperative effects. This report was also in agreement with our results.

Patient compliance and unintentional movements were strong positive and negative factors associated with dentist satisfaction, respectively (patient compliance: OR, 10.747; 95% CI, 4.851–23.812; *P*<0.001; unintentional movements: OR, 0.035; 95% CI, 0.012–0.104; *P*<0.001). Even 2 mg/kg/hr of propofol induced unintentional movements in 4.6% of the patients, while a dosage of 3 mg/kg/hr induced unintentional movements in 9.7% (Fig 5). These findings also reflect the reduction in dentist satisfaction with increasing doses of propofol (Fig 4). Therefore, propofol also exhibits an optimal peak value in terms of dentist satisfaction, if the loss of intraoperative memory in the patient and the unintentional movements of patients are both considered.

The aim of this study was to explore whether optimal doses of midazolam and propofol necessary for the achievement of high levels of satisfaction in both patients and dentists could be determined. The average weight of our patients was 59 kg, and a dose of 3 mg of midazolam corresponded to approximately 0.05 mg/kg. We previously simulated the effect-site concentration of midazolam using the Zomorodi model [9]. The peak value of midazolam was 60.8 ng/ml, decreasing to 39.3 ng/ml within 30 min of administration. This range of effect-site concentrations corresponded to the sedative effects as visualized using electroencephalography [10, 11]. The doses of midazolam used in previous studies have varied considerably, including doses of 2 mg [8], 0.03 mg/kg [12], and 8–9 mg [13]. Since the average dose that patients voluntarily administered using a patient-controlled sedation system was 1.9 mg, 2 mg might be sufficient to induce the minimum level of sedation required for dental treatments, including molar extractions [14]. Doses of over 8 mg may cause desaturation, requiring intensive monitored anesthesia care. While a study using a higher dose did not report an increased frequency of desaturation, adverse events affecting the digestive system, including nausea and vomiting (6/133 cases), were reported in a series of patients with dental anxiety [13]. A small dose of midazolam (2 mg) was reportedly sufficient to induce amnesia and anxiolytic in patients undergoing outpatient surgery [8]. Amnesia and anxiolytic may be the main purposes of using midazolam.

We also found that a higher dose of propofol increased unintentional movements (Fig 5). In contrast, a higher dose induced memory loss in most patients (82.9% at a dose of 2 mg/kg/hr, 87.8% at a dose of 3 mg/kg/hr) (Fig 3). We simulated the effect-site concentration of propofol using the Marsh model [15]. The effect-site concentration of propofol at a dose of 3 mg/kg/hr in our study was approximately 1.0 μg/ml at 20 min after administration, reaching 1.3 μg/ml at 60 min after the start of propofol administration. These values seem to agree with the findings of previous reports [16–18]. The optimal effect-site concentration of propofol has been reported to be 0.87–1.67 μg/ml in patients with spinal anesthesia [16]. Another clinical study reported that 1.5–2.5 μg/ml administered using target-controlled infusion produced a relatively better result, compared with higher doses (3.0–4.0 μg/ml). However, even the low-dose group in this previous study had an incidence of more than 10% for airway and cardiovascular events. Therefore, even 1.5–2.5 μg/ml might be too high to prevent unwanted events when propofol is used for the maintenance of sedation [17]. A report using a patient-controlled device to maintain propofol sedation showed that the patients selected a dose of 1.0–2.5 μg/ml. This report indicated that 32.5% of the patients had an oxygen saturation level of less than 95%; thus, doses higher than 2.5 μg/ml may be too high to avoid adverse effects [18].

Bennett [19] reported that a propofol dosage of 6 mg/kg/hr, inducing general anesthesia or deep sedation, was required to reduce patient movements during dentoalveolar surgery. This deep level of anesthesia also diminished unintentional movements because of the anesthesia level. However, we would like to suggest that even dosages of less than 3 mg/kg/hr might minimize patient movements. Since a lower dose is more advantageous from the perspective of not inducing desaturation, professional titration to find the optimal dose is recommended.

Monitored Anesthesia Care (MAC) is a concept that has been proposed to manage patients’ conditions safely by a professional anesthesia provider. This concept emphasizes the securement of the patient’s condition when the sedation level becomes deeper than expected. Securing a patient is the most important aspect of MAC. In addition, patient management using an optimal dose of anesthetics and sedatives without inducing any adverse events may also be important for the concept of MAC. Both patient and dentist satisfaction should also be considered during MAC, since the patients are not completely unconscious.

Another factor contributing to patient satisfaction is the duration of the procedure. Even though the patient experiences a type of blurred consciousness, he or she cannot tolerate the same position for long periods of time. Our report showed that a period of up to approximately 40 min may be a tolerable time, since the average time in the dissatisfaction group was 50 min (Table 3). The total dose of propofol was a negative factor influencing patient satisfaction, and this result might also have been related to a longer treatment period.

The present study had some limitations. Because our data was collected at a single center, our findings might not reflect the practices at other centers. Nevertheless, midazolam and propofol are commonly used sedative agents [20]. Furthermore, our study was performed in a clinical setting; therefore, patients were not randomized and the dental anesthesiologists were not blinded. These study limitations might have introduced a bias.

In conclusion, we found that 3 mg of midazolam and 3 mg/kg/hr of propofol may be the optimal doses for maximizing both patient and dentist satisfaction. Although this level of sedation is relatively light, memory loss and an absence of unintentional patient movements can be expected without adverse events.

## Supporting information

S1 Checklist TREND checklist. (PDF)

S1 Protocol Study protocol. (DOCX)

S2 Protocol Study protocol (original language version). (DOCX)
