# Supplementary material for: Optimal and safe standard doses of midazolam and propofol to achieve patient and doctor satisfaction with dental treatment: A prospective cohort study
Source: PLoS One. 2017 Feb 9;12(2):e0171627. doi: 10.1371/journal.pone.0171627 (PMC5300152; doi:10.1371/journal.pone.0171627)
Supplement: S1 Protocol — (DOCX) [file pone.0171627.s002.docx]

**Application for clinical research**

**Title:** The optimal standard dose of the intravenous sedation by using midazolam and propofol for dental procedure

1. Purpose and significance of the study

Overdoses of benzodiazepine occasionally cause agitation or lethal respiratory suppression. The sensitivity to sedatives may also vary between individuals, possibly leading to an overdose or the excessive use of multiple drugs. Our study examine whether an optimal dose could be determined and whether the required dose for individuals may vary using a statistical analysis based on data from a large cohort.

1. What to be clarified

To establish the safety of intravenous sedation for dental procedures, we tried to clarify whether the optimal regimen could be determined.

1. Reason for human trial

Our study cannot be performed in animal study.

1. Flow chart of the study ( from explanation to the end of the study)
2. Patients’ enrollment

All the participants are informed the nature of this study and the informed consent is obtained more than 1 week before the treatment. 1. Usual regimen of intravenous sedation, 2. Vital sign monitoring, 3. Interview after the procedure

1. Procedure
   1. vital sign monitoring; blood pressure, pulse oximetry every 5 minutes
   2. injection of 1 mg midazolam thorough intravenous route
   3. a dose of 1 mg of midazolam is administered at 1-minute intervals until adequate sedation
   4. propofol is then infused continuously to maintain the sedation level and sedation level is checked
   5. to evaluate amnesia effect, specific word is verbally informed
   6. treatment starts
   7. any adverse event should be appropriately responded
   8. questionnaire for doctors (Attachment 1)
   9. discharge is admitted according to the level of recovery
   10. questionnaire for patients (Attachment 2)
2. Scientific rationale of this study
   1. Design of this study

Since patients’ and doctors’ satisfaction can be subjectively evaluated, questionnaire should be the unique method to assess this objective.

- 1. Necessity of the clinical information

Questionnaires are necessary to analyze this study.

- 1. Rationale of the number of the participants

Our cases per year are 1217 cases in 2009, 1529 in 2010. Our study period is one year and half. Since we had 770 cases till May in 2011, we can expect 1,000 cases until the designated period.

1. Ethical consideration
   1. Any psychological, physiological, economic distress

Our study is performed under the normal procedure of the dental treatment using intravenous sedation. We do not expect any unwanted event. We protect patient’s privacy by means of anonymous record for analysis.

- 1. Cessation by the will of participants

We always accept patients’ will, and we smoothly stop the study in case the patients want to quit the study.

- 1. Inquiry and claim

We always accept the inquiry and claim from the patients.

- 1. Unexpected adverse event and its treatment

We have not encountered any lethal event due to intravenous sedation. We suspect respiratory depression, aspiration pneumonia, allergic reaction or dizziness. We appropriately counteract these events.

1. Intellectual asset

Any intellectual asset produced by this study resides to the study group, not to the participants.

(Attachment 1）

**Questionnaire for doctors**

(1) Did the patient open mouth smoothly?

□ Rapid (Mouth gag was unnecessary.)

□ Slow (Sometimes mouth gag was necessary.)

□ None (Mouth gag was necessary.)

(2) How was the response of the patient?

□ Rapid

□ Slow

□ None

(3) Was the patients cooperative?

□ Good

□ Fair

□ Poor

□ Bad

(4) Did you see gag reflex, coughing, or excessive movements of the patient?

□ None

□ Acceptable

□ Frequent

□ Sizable

(5) Was the patients well sedated?

□ Good

□ Fair

□ Poor

□ Bad （Reason： ）

**Thank you for your cooperation with our questionnaire.**

(Attachment 2）

**Questionnaire for patients**

(1) Do you recall a word that you have heard before treatment?

□ No (full)

□ No (with a clue)

□ Yes (but not sure)

□ Yes (full)

If you answered yes, what is it? （ ）

(2) Do you recall the treatment?

□ None

□ A little

□ Most

□ All

(3) Were you sedated well?

□ Good

□ Fair

□ Poor

□ Bad （Reason： ）

**Thank you for your cooperation with our questionnaire.**
