# Supplementary material for: Optimal and safe standard doses of midazolam and propofol to achieve patient and doctor satisfaction with dental treatment: A prospective cohort study
Source: PLoS One. 2017 Feb 9;12(2):e0171627. doi: 10.1371/journal.pone.0171627 (PMC5300152; doi:10.1371/journal.pone.0171627)
Supplement: S2 Protocol — (DOCX) [file pone.0171627.s003.docx]

**研究実施計画書**

研究課題名：ミダゾラムとプロポフォールの併用による静脈内鎮静法の至適投与量に関する研究

1　研究の意義、重要性

　局所麻酔下での手術の場合、患者の快適性を向上させるために静脈内鎮静法が適用される。歯科領域では気道と術野が同一部位という特異性があるため、患者の意識を失わせずに恐怖心や不安感のみを除去し、自主的かつ持続的に気道確保が可能で、術者の指示に対して適切に反応できるという許容範囲の狭い鎮静レベルが求められる。こういった背景から、作用時間が比較的長いが健忘効果の期待できるミダゾラムと作用時間が短いが鎮静レベルを調節しやすいプロポフォールの併用が日々の臨床で行われている。しかし、その使い方は担当医によって様々で、バイタルサインや他覚的症状を参考に鎮静レベルの評価を行い、臨床経験を基に投与量を決定しているのが現状である。本研究では、ミダゾラムとプロポフォールの使用量と健忘効果も含めた患者の満足度および治療をする術者の満足度を調べることにより、歯科領域における静脈内鎮静法の方法論を確立することを目的とする。このように、使用する薬剤の投与量と患者の呼吸抑制、歯科治療の手技的難易度との関係について解析を行うことによって、より安全で快適な静脈内鎮静法の方法を確立することは歯科医療にとって重要である。

2　この研究によって明らかにしようとしていること

歯科領域における静脈内鎮静法を理論的に構築し、より安全で快適な方法論を確立する。

3　ヒトで研究を行わなければならない理由

　本研究では有効性については健忘効果、患者の快適性と歯科治療の手技的難易度の3項目を指標とし、安全性についてはバイタルサインとリカバリー時間の2項目を指標として各々評価するため、ヒトを対象としない代替え法は不可能である。

4　研究のフローチャート（患者への説明から参加終了まで）

1)　患者の選択

・静脈内鎮静法を行うことが決定した時点で本研究の候補者とし、参加に同意した患者を対象とする。

・本研究参加に関する同意については、①検査方法や鎮静剤の投与量、投与方法は通常どおりに行う

　こと②検査中にバイタルサインのモニタリングをすること③帰宅前に治療時の記憶について聞き取

　り調査をすることの3点を、鎮静法施行の1週間以上前に歯科外来、病棟あるいは手術室で説明

　し書面で同意を得ておく。

2)　方法

1. 患者が診療台に座った後に、麻酔科医が自動血圧計とパルスオキシメータをそれぞれ患者の左右上肢に装着する。バイタルサインとして血圧、脈拍数、SpO_2_を測定し、これを基準値とする。その後、鎮静法終了まで5分間隔で測定記録する。
2. 麻酔科医は静脈路を確保し，ミダゾラムを目標鎮静レベルまで1mgずつゆっくり投与する。
3. ミダゾラム投与後，プロポフォール持続投与開始する（年齢・体重により適宜増減）。治療開始前，治療終了直後の鎮静レベルをチェックする。術中は治療中にだいたいどの位で推移したかで判定する。
4. プロポフォール開始後、健忘効果の指標とするため患者に「言葉」を覚えてもらう（術後に覚えているか確認する）。
5. 治療医に局所麻酔あるいは治療を開始してもらう。
6. 特記事象があった場合には対処する（別紙添付）。
7. 治療終了後、患者の協力度、あるいは咽頭・咳反射や体動の状態から治療の手技的難易度を治療医にアンケート形式で評価してもらう（別紙添付）。
8. 鎮静薬投与終了から帰宅可能と判断されるまでの時間をリカバリー時間とする。
9. 帰宅前に問診を行い、健忘効果、快適性についての情報を得た後、参加終了とする（別紙添付）。

5　実施計画の科学的妥当性、有効性

1)　研究方法の妥当性

　　本研究は患者の記憶や満足度を調査する研究であるのでヒトを対象とした臨床研究となる。この調査は患者の主観的な評価が必要であるため問診による本研究計画は科学的に妥当と考えられる。

2)　使用する生体試料や臨床情報の必要性

　　本研究は患者の主観的な評価が必要であるため、問診で得られた情報は解析に必須である。

3)　組込予定数算出の科学的根拠と実現性

　　2種類の薬剤の投与量はそれぞれ連続数であり、その組み合わせは多様である。患者の満足度も個人差が多く多様である。したがって、多くの症例数が必要となる。本研究は観察研究であり、研究期間に可及的に多くの症例を集めることがデータの信頼性を向上させる。

　実現性の根拠としては、静脈内鎮静法の数は年々増加傾向にあり、平成21年1217例、平成22年1529例、平成23年においても5月までで770例の症例数があり、1年半で1000例というのは充分に確保できる数字と考える。

6　倫理的配慮

1)　参加者の予想される精神的、肉体的、経済的、時間的な苦痛や負荷

　　本研究では通常の検査および治療目的の範囲を越えず、それによって患者が大きな不利益が生じることはない。結果が外部に漏れた場合は将来、様々な不利益を被る可能性がある。これを防ぐためプライバシーと人権の擁護には、提供された臨床情報を連結可能匿名化とし、最大限の配慮を行う。

2)　参加の中断に対する対応

　　本研究への参加は対象者の自由意思により決定される。また、同意後でも治療の開始の有無に関わらずいつでも撤回でき、同意しない場合においても治療内容も含めいかなる不利益を被ることもない。

3)　問い合わせ、苦情に対する対応

　「ご協力のお願い」に電話と電子メールによる問い合わせ方法を明記した。

4)　研究期間中の不測の事態に対する対応

　静脈内鎮静法が必要な患者に対して適切な方法で管理するため、研究に参加したことによる直接的な不利益はない。今のところ致命的となった例の報告はないが、静脈麻酔薬による副作用として呼吸抑制、誤嚥性肺炎、アレルギー、帰宅時のふらつき等の副作用の発現も予想される。万一この方法によって健康被害が生じた場合、特別な補償の制度はないが誠意をもって治療にあたる。

7　研究から生じる知的財産権について

この研究から経済的利益が生じた場合、その権利は研究を実施する研究機関や研究者に属し，資料提供者がこの権利を持つことはない。

**アンケートのご協力よろしくお願いします** （先生用）

**質問１**　患者さんの開口はどうでしたか？どれか1つにだけ○を付けて下さい

1 指示によって即座に開口でき，開口を保持できる．開口器は必要ない

2 指示によって緩慢に開口し，放っておくと閉口してしまう．場合によっては開口器が必要となる

3 指示によって開口しない．開口器が必須

**質問２**　患者さんの応答はどうでしたか？どれか1つにだけ○を付けて下さい

1 速やか

2 ゆっくり

3 全くなし

**質問３**　患者さんの協力度はどうでしたか？どれか1つにだけ○を付けて下さい

1 良好だった

2 やや良好だった

3 やや不良だった

4 不良だった

**質問４**　患者さんの咽頭反射・咳・体動はいかがでしたか？どれか1つにだけ○を付けて下さい

1 ほぼ皆無だった

2 許容範囲内だった

3 やや多かった

4 かなり多かった

質問５　鎮静法はいかがでしたか？どれか1つにだけ○を付けて下さい

1 円滑に治療できた

2 支障なく治療できた

3 治療にやや支障があった

4 治療にかなり支障があった（理由： ）

**アンケートのご協力よろしくお願いします** （患者用）

**質問１**　治療前に覚えて頂いたものを覚えていますか？どれか1つにだけ○を付けて下さい

1 全く覚えていない．ヒントを聞いても全く思い出せない

2 何となく覚えている．ヒントを聞いても思い出せなかった

3 何となく覚えている．ヒントを聞いて思い出せた

4 覚えている．ヒントを聞かなくても思い出せる

**※思い出せた方に質問します**

　それは何でしたか？（ ）

**質問２**　治療中のことを何か覚えていますか？どれか1つにだけ○を付けて下さい

1 全体を通して覚えていない，思い出せない

2 ほとんど覚えていないが，一部思い出せる

3 ほとんど覚えているが，一部思い出せない

4 全体を通して覚えている，思い出せる

**質問３**　鎮静法はいかがでしたか？どれか1つにだけ○を付けて下さい

1 快適に治療を受けることができた

2 支障なく治療を受けることができた

3 治療中やや不快だった

4 治療中かなり不快だった（理由： ）
